# A comparison of the clinical outcomes of patients with invasive lobular carcinoma and invasive ductal carcinoma of the breast according to molecular subtype in a Korean population

**DOI:** 10.1186/1477-7819-12-56

**Published:** 2014-03-13

**Authors:** Seung Taek Lim, Jong Han Yu, Heung Kyu Park, Byung In Moon, Byung Kyun Ko, Young Jin Suh

**Affiliations:** 1 Department of Surgery, St. Vincent’s Hospital, The Catholic University of Korea, 93, Joongboo-Daero Suwon, Paldal-gu, Kyunggi-do 442-723, Korea; 2 Department of Surgery, Asan Medical Center, University of Ulsan College of Medicine, 388-1, Poongnap-dong, Songpa-gu, Seoul 138-736, Korea; 3 Department of Surgery, GachonUniversityGillHospital, Gachon University College of Medicine, 1198, Guwall-dong, Namdong-gu, Incheon 405-760, Korea; 4 Department of Surgery, Ewha University Mokdong Hospital, EwhaWomans University College of Medicine, 1071 Anyangcheon-ro, Yangcheon-gu, Seoul 158-710 Korea; 5 Department of Surgery, Ulsan University Hospital, University of Ulsan College of Medicine, 877 Ulsan 682-714 Korea; 6 Department of Surgery, Division of Breast and Thyroid Surgical Oncology, St. Vincent’s Hospital, The Catholic University of Korea, 93, Joongboo-Daero Suwon, Paldal-gu, Kyounggi-do 442-723, Korea

**Keywords:** Invasive lobular carcinoma, Invasive ductal carcinoma, Molecular subtype, Breast cancer-specific survival, Overall survival

## Abstract

**Background:**

To investigate the clinicopathological characteristics and the survival outcomes of invasive lobular carcinoma (ILC) patients compared to invasive ductal carcinoma (IDC) patients according to their molecular subtype.

**Methods:**

We compared the clinicopathological characteristics, breast cancer-specific survival (BCSS) and overall survival (OS) between patients with IDC (n = 14,547) and ILC (n = 528).

**Results:**

The ILC presented with a larger tumor size, more advanced cancer stage, increased rate of hormonal receptor positivity, human epidermal growth factor 2 (HER2) negativity and mastectomy than the IDC. The ILC patients more frequently presented with the luminal A subtype, whereas the IDC patients more frequently presented with the luminal B, HER2-overexpression, or triple negative subtype. The BCSS and OS were not significantly different between the IDC and ILC for each molecular subtype.

**Conclusions:**

Similar to IDC patients, molecular subtype should be considered when determining the prognosis and treatment regimen for ILC patients.

## Background

Invasive lobular carcinoma (ILC), also known as infiltrating lobular carcinoma, is the second most frequent histological subtype of breast cancer. It was first described by Foote and Stewart in 1941 and it is found in approximately 5 to 15% of patients in western countries [1-4]. The incidence rate of ILC has steadily increased over the last 20 years [5].

In the past, the prognosis of ILC compared with invasive ductal carcinoma (IDC) has been controversial [6-10]. Although ILC has been reported as more multifocal and bilateral than IDC [11-14], IDC and ILC present with similar clinical manifestations. Moreover, the treatment strategies for IDC and ILC are similarly based on TNM staging.

The importance of molecular subtype in the treatment and prognosis of IDC has been increasingly emphasized in the recent literature. However, relatively little is known aboutILC despite its increasing incidence. Moreover, reports on the prognostic significance of the molecular subtypes of ILC in Asia are limited because there is a lower incidence of ILC in Asia compared to western countries [15,16].

Therefore, in this study, we aimed to compare the association between the molecular subtype and the clinical outcomes of IDC and ILC in Korea using patient information from the nationwide Korean Breast Cancer Registry (KBCR) database. The primary objective of this investigation was to compare the survival outcomes of IDC and ILC according to molecular subtype. The second objective was to determine the association between various clinicopathological factors and survival outcomes.

## Methods

### Korean breast cancer registry

The KBCR database is a nationwide database that includes 41 university hospitals and 61 surgical training hospitals [17]. This database provides information pertaining to patient survival, sex, age, the surgical method used, the stage of cancer based on the seventh American Joint Committee on Cancer (AJCC) classification, the pathological characteristics of the patient’s tumor, and any adjuvant treatment received.

### Study population

All female breast cancer patients who were listed in the KBCR and were diagnosed between January 1995 and December 2006 were selected for this study. Clinicopathological data, including age, date of surgery, method of surgery, tumor size at presentation, axillary lymph node status, TNM stage andlymphovascular invasion were collected. Immunohistochemical results evaluating the expression of the estrogen receptor (ER), progesterone receptor (PR), and human epidermal growth factor receptor 2(HER2) were also collected. A patients was considered ER and PR positive if 10% or more of their tumor was positively stained. For HER2, an immunohistochemical staining score of 3+ was considered positive. Because fluorescence *in situ* hybridization (FISH) was unavailable during most of the study period, a HER2 score of 2+ was considered negative.

All of the patients at risk for relapse received adjuvant chemotherapy followed by local radiotherapy and/or hormonal therapy according to the recommended therapeutic regimen at the time of surgery as determined by international guidelines, such as the national comprehensive cancer network (NCCN).

Patients were excluded if they had metastatic disease at the time of presentation, bilateral breast cancer, a history of previous malignancy, or had received neoadjuvant chemotherapy. We also excluded patients who were not treated with a curative intent (no surgery, no axillary staging, or had tumor tissue remaining after their final surgery), patients without follow-up data, and patients whose ER, PR, HER2, and lymphovascular invasion status was unknown.

### Statistical analysis

Molecular subtype was categorized as follows: luminal A(LA; ER + and/or PR+, and HER2-), luminal B(LB; ER + and/or PR+, and HER2+), HER2+ (HER2; ER- and PR-, and HER2+), and triple negative (TN; ER- and PR-, and HER2-).

Breast cancer-specific survival (BCSS) was defined as the time from the date of breast cancer diagnosis until the date of breast cancer-related death or the date of the last follow-up. Overall survival (OS) was defined as the time from the date of breast cancer diagnosis until the date of death (from any cause) or the date of the last follow-up.

Characteristic differences between the IDC and ILC groups were compared using independent *t*-test and chi-square analyses, as appropriate. Survival curves were obtained using the Kaplan-Meier method, and the survival curves were compared using the log rank test. Multivariate Cox proportional hazard regression analysis was used to assess the independent prognostic significance of various clinical and histopathological characteristics of the tumors. All of the statistical analyses were performed using SPSS version 11.0 (SPSS, Chicago, IL,USA).

## Results

### Patients’ characteristics and distribution based on molecular subtype

A total of 41,813 patients diagnosed with breast cancer between 1995 and 2006, whose information was available in the KBCR database, were selected for this study. After exclusion, we identified 15,075 invasive breast cancer patients. Of the 15,075 patients in the study population, 14,547 (96.5%) presented with IDC and 528 (3.5%) presented with ILC. The clinical, demographic, and treatment features of the patients in the study population are summarized in Table 1.

**Table 1:** Baseline patient characteristics according to invasive ductal and invasive lobular histological subtype

	**IDC group (n = 14,547)**	**ILC group (n = 528)**	***P*-value**
Age			
Mean ± SD	48.5 ± 10.3	48.9 ± 9.3	0.297
Median (range)	47.0 (19.0 to 93.0)	47.0 (24.0 to 82.0)	0.321
<50	8736 (60.1)	323 (61.2)	0.109
50≤	5811 (39.9)	205 (38.8)	
Tumor size			
Mean ± SD	2.3 ± 1.4	2.9 ± 1.9	<0.001
Median (range)	2.0 (0.1 to 16.0)	2.5 (0.3 to 14.0)	<0.001
T ≤ 2 cm	7,556 (51.9)	218 (41.3)	<0.001
2 cm < T ≤ 5 cm	6,341 (43.6)	251 (47.5)	
5 cm < T	650 (5.2)	59 (11.2)	
Nodal status			
0	8238 (56.6)	289 (54.7)	0.248
1 to 3	4067 (28.0)	141 (26.7)	
4 to 9	1481 (10.2)	67 (12.7)	
10≤	761 (5.2)	31 (5.9)	
TNMstage			
stage I	5323 (36.6)	166 (31.4)	0.001
stage II	6819 (46.9)	244 (46.2)	
stage III	2405 (16.5)	118 (22.4)	
Lymphatic invasion			
no	9,337 (64.2)	343 (65.0)	0.714
yes	5,210 (35.8)	185 (35.0)	
Vascular invasion			
no	11,794 (81.1)	387 (73.3)	<0.001
yes	2,753 (18.9)	141 (26.7)	
Estrogen receptor status			
negative	5,698 (39.2)	103 (19.5)	<0.001
positive	8,849 (60.8)	425 (80.5)	
Progesterone receptor status			
negative	6,370 (43.8)	134 (25.4)	<0.001
positive	8,177 (56.2)	394 (74.6)	
HER2			
negative	11,381 (78.2)	496 (93.9)	<0.001
positive	3,166 (21.8)	32 (6.1)	
Radiation therapy			
no	6,570 (45.2)	265 (50.2)	0.023
yes	7,977 (54.8)	263 (49.8)	
Chemotherapy			
no	2,499 (17.2)	96 (18.2)	0.549
yes	12,048 (82.8)	432 (81.8)	
Hormonal therapy			
no	4,549 (31.3)	81 (15.3)	<0.001
yes	9,998 (68.7)	447 (84.7)	
Surgery			
mastectomy	8,112 (55.8)	354 (67.1)	<0.001
breast conserving surgery	6,435 (44.2)	174 (32.9)	
Molecular subtype			
luminal A	8,196 (56.3)	439 (83.2)	<0.001
luminal B	1,638 (11.3)	25 (4.7)	
HER2-overexpression	1,528 (10.5)	7 (1.3)	
Triple negative	3,185 (21.9)	57 (10.8)	

Data are express as the n (%), means ± SD and median (range).

HER2, human epidermal growth factor receptor 2; IDC, invasive ductal carcinoma; ILC, invasive lobular carcinoma.

The ILC patients presented with larger (*P* < 0.001) and more advanced stage (*P* = 0.001) tumors. The rate of hormone receptor positivity (*P* < 0.001) and HER2 negativity (*P* < 0.001) was increased in the ILC patients, as was the rate of mastectomy (*P* < 0.001). Statistically significant differences in the percentage of patients receiving postoperative external radiotherapy might be explained by the reduced percentage of ILC patients receiving breast-conserving operations (Table 1).

Table 1 also shows the distribution of the IDC and ILC patients based on their molecular subtypes. Whereas the ILC patients more frequently presented with the luminal A subtype, the IDC patients more frequently presented with either the luminal B, HER2-overexpression, or triple negative subtype. The differences in the molecular subtypes between the IDC and ILC patients were statistically significant (*P* < 0.001).

### Breast cancer-specific survival and overall survival of the IDC and ILC patients

**Figure 1:**
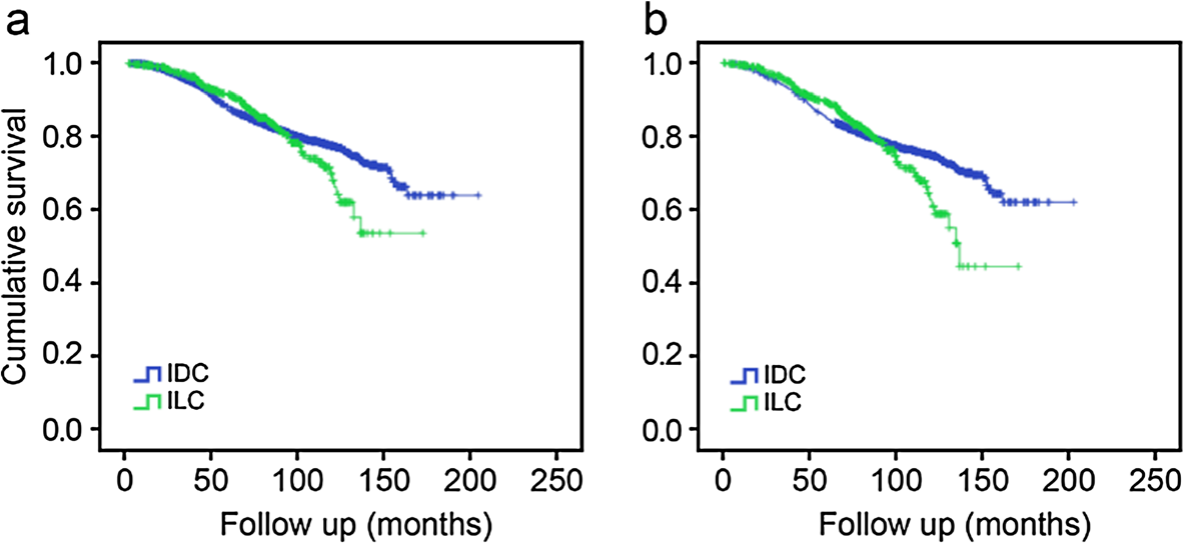
Breast cancer-specific survival (a) and overall survival (b) according to histological subtype.

The median follow-up period was 81.91 months, 82.37 months, and 69.41 months for the total patient populations, the patients with IDC, and the patients with ILC, respectively. Figure 1 shows the survival curves for the IDC and ILC cohorts. The BCSS (Figure 1a, *P* = 0.500) and OS (Figure 1b, *P* = 0.503) were not significantly different between these two groups.

### Effect of molecular subtype on the breast cancer-specific survival and overall survival of IDC and ILC patients

**Figure 2:**
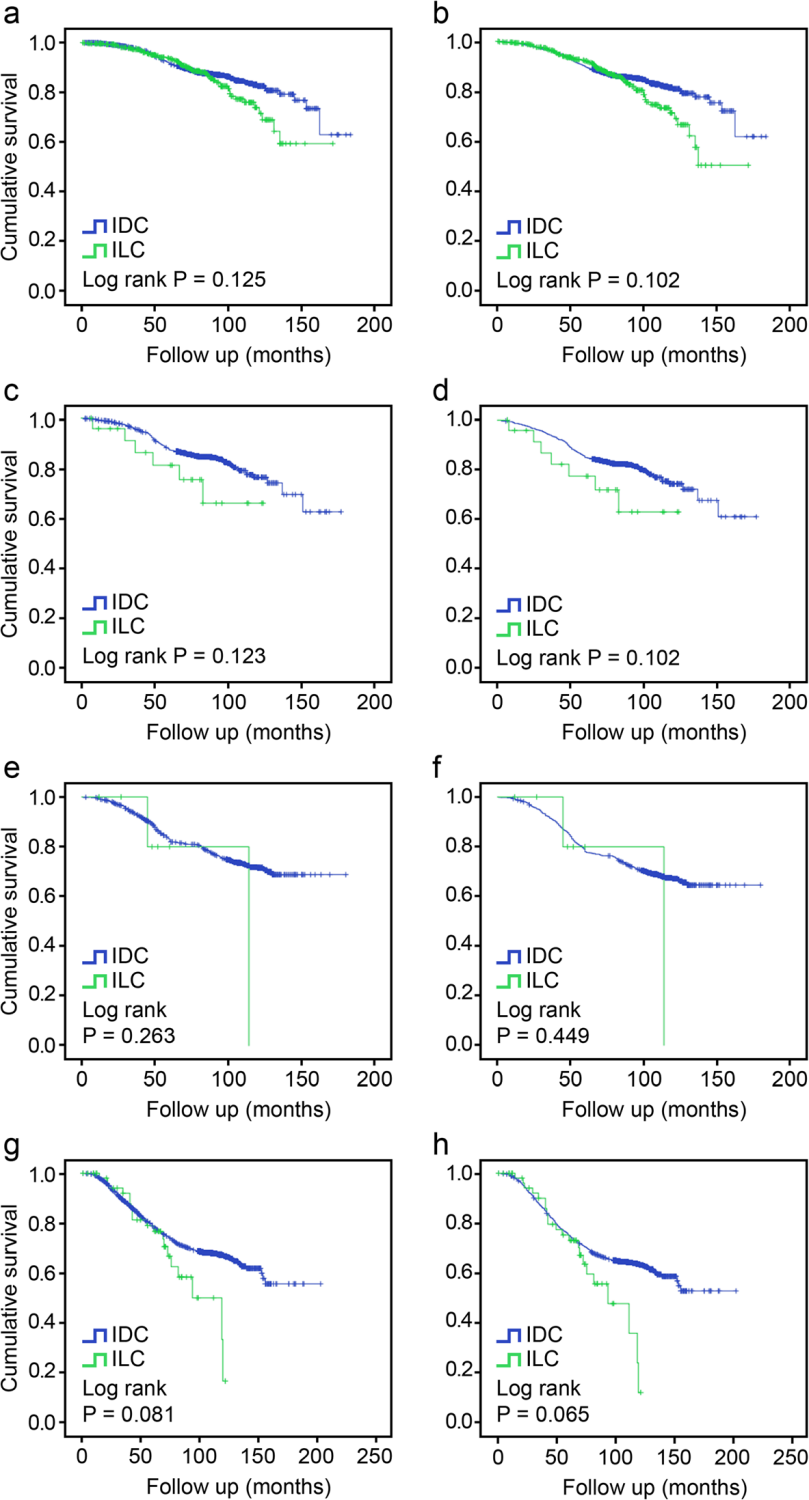
BCSS and OS according to molecular subtype: luminal A(a,b), luminal B(c,d), HER2-overexpression (e,f), and triple negative (g,h).

Figure 2 shows the impact of molecular subtype on breast cancer-specific and overall survival in the IDC and ILC patients. The BCSS rates according to molecular subtype were 88.2% IDC versus 87.0% ILC for luminal A (*P* = 0.126), 84.3% IDC versus 76.0% ILC for luminal B (*P* = 0.130), 73.8% IDC versus 71.4% ILC for HER2 (*P* = 0.276), and 68.3% IDC versus 66.7% ILC for TN (*P* = 0.084). Although the triple negative ILC patients tended to exhibit poorer outcomes compared with the triple negative IDC patients (*P* = 0.084), we failed to find a statistically significant difference in BCSS between the IDC and ILC patients in terms of the luminal A, luminal B, HER2-overexpression, and TN subtypes. Similarly, there was no statistically significant difference in OS between the IDC and ILC patients with respect to molecular subtype.

### Multivariate analysis for prognostic factors

A multivariate survival analysis was performed using a Cox regression model to determine the independent prognostic factors for BCSS and OS.

For the BCSS of the IDC group, several variables were found to be independent prognostic factors: a tumor diameter of larger than 2 cm at the time of diagnosis (2 cm < T ≤ 5; HR = 1.228; 95% CI, 1.101 to 1.369; *P* < 0.001/5 cm < T; HR = 1.434; 95% CI, 1.218 to 1.689; *P* <0.001); stage III disease (HR = 1.563; 95% CI, 1.344 to 1.818; *P* < 0.001); lymphatic invasion (HR = 4.206; 95% CI, 3.748 to 4.721; *P* < 0.001), vascular invasion (HR = 3.019; 95% CI, 2.747 to 3.317; *P* < 0.001), ER positivity (HR = 0.736; 95% CI, 0.633 to 0.857; *P* < 0.001), HER2 positivity (HR = 1.426; 95% CI, 1.200 to 1.695; *P* < 0.001); and molecular subtype (LB; HR = 1.175; 95% CI, 1.022 to 1.350; *P* = 0.023/HER2; HR = 1.426; 95% CI, 1.200 to 1.695; *P* < 0.001/TN; HR = 2.523; 95% CI, 2.160 to 2.948; *P* < 0.001) (Table 2). In terms of the OS of the IDC group, a tumor diameter of larger than 2 cm at the time of diagnosis (2 cm < T ≤ 5; HR = 1.233; 95% CI, 1.112 to 1.366; *P* < 0.001/5 cm < T; HR = 1.395; 95% CI, 1.192 to 1.632; *P* < 0.001), stage III disease (HR = 1.392; 95% CI, 1.211 to1.601; *P* < 0.001), lymphatic invasion (HR = 3.212; 95% CI, 2.899 to 3.559; *P* < 0.001), vascular invasion (HR = 2.777; 95% CI, 2.540 to 3.036; *P* < 0.001), ER positivity (HR = 0.671; 95% CI, 0.585 to 0.711; *P* < 0.001), HER2 positivity (HR = 1.401; 95% CI, 1.197 to 1.640; *P* < 0.001), and molecular subtype (LB; HR = 1.182; 95% CI, 1.093 to 1.344; *P* = 0.011/ HER2; HR = 1.406; 95% CI, 1.201 to 1.646; *P* < 0.001/ TN; HR = 2.219; 95% CI, 1.924 to 2.559; *P* < 0.001) were found to be significant independent prognostic factors (Table 2). In terms of the BCSS of the ILC group, stage III disease (HR = 4.242; 95% CI, 2.023 to 8.897; *P* < 0.001), and the triple negative subtype (HR = 3.543; 95% CI, 2.078 to 6.042; *P* < 0.001) were significantly associated with prognosis (Table 3). Similarly, stage III disease (HR = 3.647; 95% CI, 1.826 to 7.285; *P* < 0.001), and the triple negative subtype (HR = 3.977; 95% CI, 1.653 to 9.565; *P* < 0.001) were significantly associated with OS in the ILC group (Table 3).

**Table 2:** Results of the Cox regression analysis evaluating the breast cancer-specific survival (BCSS) and overall survival (OS) of patients with invasive ductal carcinoma (IDC)

	**IDC (n = 14,547)**			
	**BCSSHR (95% CI)**	***P*-value**	**OS HR (95% CI)**	***P*-value**
Age				
<50	1		1	
50≤	1.060 (0.929 to 1210)	0.386	1.067 (0.944 to 1.206)	0.302
Surgery				
mastectomy	1		1	
breast conserving surgery	1.049 (0.937 to 1.175)	0.405	1.08 (0.906 to 1.121)	0.882
Tumor size				
T ≤ 2 cm	1		1	
2 cm < T ≤ 5 cm	1.228 (1.101 to 1.369)	<0.001	1.233 (1.112 to 1.366)	<0.001
5 cm < T	1.434 (1.218 to 1.689)	<0.001	1.395 (1.192 to 1.632)	<0.001
Nodal status				
0	1		1	
1 to 3	1.045 (0.919 to 1.189)	0.501	1.043 (0.926 to 1.174)	0.488
4 to 9	0.940 (0.679 to 1.302)	0.710	0.903 (0.662 to 1.233)	0.522
10≤	1.018 (0.734 to 1.412)	0.916	0.951 (0.695 to 1.30)	0752
TNMstage				
stage I	1		1	
stage II	1.090 (0.949 to 1.253)	0.223	1.018 (0.897 to 1.155)	0.784
stage III	1.563 (1.344 to 1.818)	<0.001	1.392 (1.211 to 1.601)	<0.001
Molecular subtype				
luminal A	1		1	
luminal B	1.175 (1.022 to 1.350)	0.023	1.182 (1.039 to 1.344)	0.011
HER2-overexpression	1.426 (1.200 to 1.695)	<0.001	1.406 (1.201 to 1.646)	<0.001
Triple negative	2.523 (2.160 to 2.948)	<0.001	2.219 (1.924 to 2.559)	<0.001
Lymphatic invasion				
no	1		1	
yes	4.206 (3.748 to 4.721)	<0.001	3.212 (2.899 to 3.559)	<0.001
Vascular invasion				
no	1		1	
yes	3.019 (2.747 to 3.317)	<0.001	2.777 (2.540 to 3.036)	<0.001
Estrogen receptor status				
negative	1		1	
positive	0.736 (0.633 to 0.857)	<0.001	0.671 (0.585 to 0.771)	<0.001
Progesterone receptor status				
negative	1		1	
positive	0.960 (0.826 to 1.117)	0.600	0.993 (0.861 to 1.146)	0.928
HER2				
negative	1		1	
positive	1.426 (1.200 to 1.695)	<0.001	1.401 (1.197 to 1.640)	<0.001
Radiation therapy				
no	1		1	
yes	1.012 (0.911 to 1.124)	0.826	1.010 (0.915 to 1.116)	0.841
Chemotherapy				
no	1		1	
yes	1.059 (0.895 to 1.252)	0.506	1.113 (0.958 to 1.295)	0.162
Hormonal therapy				
no	1		1	
yes	1.025 (0.914 to 1.149)	0.671	1.007 (0.905 to 1.121)	0.894

BCSS, breast cancer specific survival; HER2. human epidermal growth 2;HR, hazard ratio; IDC, invasive ductal carcinoma; OS, overall survival.

**Table 3:** Results of the Cox regression analysis evaluating the breast cancer-specific survival (BCSS) and overall survival (OS) of patients with invasive lobular carcinoma (ILC)

	**ILC (n = 528)**			
	**BCSS HR (95% CI)**	***P*-value**	**OS HR (95% CI)**	***P*-value**
Age				
<50	1		1	
50≤	0.898 (0.424 to 1.904)	0.780	0.708 (0.350 to 1.435)	0.338
Surgery				
mastectomy	1		1	
breast conserving surgery	1.059 (0.520 to 2.157)	0.874	1.224 (0.645 to 2.324)	0.536
Tumor size				
T ≤ 2 cm	1		1	
2 cm < T ≤ 5 cm	0.524 (0.205 to 1.337)	0.176	0.507 (0.215 to 1.196)	0.121
5 cm < T	0.275 (0.055 to 1.367)	0.114	0.343 (0.077 to 1.527)	0.160
Nodal status				
0	1		1	
1 to 3	0.156 (0.216 to 1.232)	0.136	0.525 (0.244 to 1.128)	0.099
4 to 9	0.307 (0.074 to 1.267)	0.103	0.335 (0.090 to 1.250)	0.104
10≤	0.441 (0.103 to 1.886)	0.270	0.449 (0.116 to 1.737)	0.246
TNMstage				
stage I	1		1	
stage II	1.271 (0.634 to 2.548)	0.500	1.409 (0.753 to 2.639)	0.284
stage III	4.242 (2.023 to 8.897)	<0.001	3.647 (1.826 to 7.285)	<0.001
Molecular subtype				
luminal A	1		1	
luminal B	2.117 (0.903 to 4.961)	0.084	2.157 (0.977 to 4.764)	0.057
HER2-overexpression	1.499 (0.349 to 6.434)	0.586	1.769 (0.344 to 9.106)	0.495
Triple negative	3.543 (2.078 to 6.042)	<0.001	3.977 (1.653 to 9.565)	0.002
Lymphatic invasion				
no	1		1	
yes	0.732 (0.389 to 1.375)	0.332	0.802 (0.455 to 1.415)	0.446
Vascular invasion				
no	1		1	
yes	1.260 (0.690 to 0.299)	0.452	1.068 (0.608 to 1.875)	0.819
Estrogen receptor status				
negative	1		1	
positive	1.649 (0.464 to 5.859)	0.439	1.657 (0.482 to 5.697)	0.423
Progesterone receptor status				
negative	1		1	
positive	0.546 (0.277 to 1.076)	0.080	0.568 (0.318 to 1.014)	0.056
HER2				
negative	1		1	
positive	3.591 (0.353 to 36.498)	0.280	3.644 (0.395 to 33.657)	0.254
Radiation therapy				
no	1		1	
yes	0.844 (0.463 to 1.537)	0.579	0.795 (0.456 to 1.387)	0.419
Chemotherapy				
no	1		1	
yes	1.178 (0.466 to 2.977)	0.730	1.011 (0.453 to 2.259)	0.979
Hormonal therapy				
no	1		1	
yes	2.063 (0.826 to 5.153)	0.121	2.112 (0.952 to 4.688)	0.066

BCSS, breast cancer-specific survival; HER2. human epidermal growth factor 2;HR, hazard ratio; ILC, invasive lobular carcinoma; OS, overall survival.

## Discussion

This study compared the clinicopathological characteristics and survival outcomes of patients with ILC and IDC according to their molecular subtype in a Korean population. Several studies have reported the relatively lower incidence rate of ILC cases in Asia compared to western countries [15,16]. In a study by Ko *et al*. [15], the incidence rate of ILC among the whole breast cancer population was reported to be 2 to 4% in Korea. Consistent with previous reports, the incidence rate of ILC was 3.5% in our study [15,16].

Our study show that ILC is associated with increased tumor size, advanced TNM stage, hormone receptor positivity, HER2 negativity, and increased mastectomy rate compared to patients with IDC. Among these characteristics, increased tumor size and advanced staging are generally accepted as poor prognostic factors. Therefore, discrepancies between OS and BCSS for the IDC and ILC cases are expected. Although it is not possible to determine precisely why the IDC and ILC cases exhibited similar patterns of OS and BCSS, despite not sharing any known prognostic markers, a reduction in HER2 positivity and an increase in the percentage of patients with the luminal type A subtype may contribute to the relatively favorable prognosis of ILC cases. However, stage III cancer status and the TN molecular subtype were found to be correlated with a decreased OS and BCSS in the ILC cases. Thus, attending physicians should discuss these matters with their patients when deciding on adjuvant therapy.

Some studies have shown that compared to IDC patients, ILC patients present with larger tumors at the time of diagnosis [11,18-20]. Consistent with these reports, the median tumor size in our study was higher in the ILC group (2.5 cm) compared to the IDC group (2.0 cm). One possible explanation for these results is the indistinct growth pattern of these tumors, which renders ILC unclear and sometimes invisible in clinical and mammographic investigations [21-23]. This unique characteristic of ILC might contribute to its late diagnosis and, consequently, to the increased size and TNM staging of the tumor at the time of diagnosis. However, despite conflicting reports regarding the degree of lymph node positivity in ILC patients compared to patients with IDC [6,23,24], we found no significant differences in lymph node positivity between the analyzed IDC and ILC cases.

In this study, the incidence of hormone receptor positivity was significantly increased in the patients with ILC compared to the patients with IDC, which is consistent with previous reports [24-26]. We also confirmed an increased incidence of the luminal subtype in the ILC patients compared to the IDC patients. In addition, HER2 positivity was significantly lower in the ILC patients compared to the IDC patients, which is consistent with previous reports showing an increased incidence of HER2- tumors in patients with ILC compared to patients with IDC [11,26,27]. However, because we considered a HER2 score of 2+ to be HER2- due to a lack of FISH amplification information for the study period, we cannot exclude the possibility that the HER2 positivity was underestimated, which would influence the incidence of both the luminal B and HER2-overexpression subtypes. However, it has been shown in various studies that approximately 25 to 50% of HER2 2+ tumors are positive by FISH amplification [28,29], and this value may provide a better estimation of the molecular subtype distribution in this study population.

Mastectomy was performed more often for the ILC patients than the IDC patients. This trend is likely due to the larger size of ILC tumors as well as their multifocality compared to IDC tumors. In addition, because the rate of breast conservation surgery is reduced in ILC patients, postoperative radiation therapy was performed less frequently in these patients, which is consistent with previous reports [20,24]. Additionally, some reports have suggested a relatively higher rate of multifocality and multicentricity for ILC tumors compared to IDC tumors, which may influence the increased rate of mastectomy in these patients [21,26]. However, we could not investigate the association between these factors because the relevant information was not available.

There are many conflicting reports regarding the prognostic differences between ILC and IDC. Pestalozzi *et al*. [1] reported a poorer survival outcome for ILC than for IDC, while many reports have suggested a similar or more favorable survival outcome for ILC patients compared with IDC patients [30-34]. In this study, we demonstrated that the survival outcome for the ILC patients was similar to that of the IDC patients. Furthermore, although the triple negative cohort of ILC patients tended to exhibit a worse survival outcome than the same cohort of IDC patients, we also found similar survival outcomes between the IDC and ILC patients of each molecular subtype. Accordingly, similar to IDC, we conclude that the molecular subtype classification should be considered as an important prognostic indicator for ILC patients. Moreover, a recent subgroup analysis of the HERA trial revealed the beneficial effects of trastuzumab therapy on survival in HER2 + ILC patients [35], which reflects the importance of incorporating molecular subtype classification into the therapeutic treatment of ILC to produce a better clinical outcome.

To our knowledge, this is the first report comparing the differences in survival outcome between IDC and ILC according to molecular subtype within an Asian population. However, our study has several limitations. First, the study design is retrospective; therefore, a risk of selection bias is present. Second, due to the lack of information and the need of a consistent protocol for determining positivity in immunohistochemical staining, we could not apply Ki-67 values for the classification of the luminal subtypes. Finally, the sample sizes of certain ILC subgroups are relatively small, and a larger study is warranted for this type of analysis.

## Conclusion

In conclusion, despite some characteristic differences, our study demonstrated a similar survival outcome for ILC patients among all molecular subtypes compared to IDC patients. Although studies with a larger sample size and a longer follow-up period should be performed to confirm our results, this study indicates that similar to IDC patients, molecular subtype should be considered for prognostic prediction and treatment decisions for ILC patients.

## Abbreviations

AJCC: American Joint Committee on Cancer; BCSS: breast cancer-specific survival; ER: estrogen receptor; FISH: fluorescence *in situ* hybridization; HER: human epidermal growth factor receptor; IDC: invasive ductal carcinoma; ILC: invasive lobular carcinoma; KBCR: Korean Breast Cancer Registry; LA: luminal A; LB: luminal B; NCCN: National Comprehensive Cancer Network; OS: overall survival; PR: progesterone receptor; TN: triple negative.

## Competing interests

There is no conflict of interest regarding this study

## Authors’ contributions

YJS and STL carried out the study conception and design. JHY, HKP, BIM and BKK were responsible for data collecting and manuscript writing. STL participated in the design of the study and performed the statistical analysis. All authors read and approved the final manuscript.
